# PIWIL1 Drives Chemoresistance in Multiple Myeloma by Modulating Mitophagy and the Myeloma Stem Cell Population

**DOI:** 10.3389/fonc.2021.783583

**Published:** 2022-01-10

**Authors:** Yajun Wang, Lan Yao, Yao Teng, Hua Yin, Qiuling Wu

**Affiliations:** Institute of Hematology, Union Hospital, Tongji Medical College, Huazhong University of Science and Technology, Wuhan, China

**Keywords:** PIWIL1, drug resistance, mitophagy, cancer stem cells (CSCs), multiple myeloma

## Abstract

As an important member of the Argonaute protein family, PIWI-like protein 1 (PIWIL1) plays a key role in tumor cell viability. However, the exact function of PIWIL1 in multiple myeloma (MM) and the underlying mechanism remain unclear. Here, we revealed that PIWIL1 was highly expressed in myeloma cell lines and newly diagnosed MM patients, and that its expression was notably higher in refractory/relapsed MM patients. PIWIL1 promoted the proliferation of MM cells and conferred resistance to chemotherapeutic agents both *in vitro* and *in vivo*. More importantly, PIWIL1 enhanced the formation of autophagosomes, especially mitophagosomes, by disrupting mitochondrial calcium signaling and modulating mitophagy-related canonical PINK1/Parkin pathway protein components. Mitophagy/autophagy inhibitors overcome PIWIL1-induced chemoresistance. In addition, PIWIL1 overexpression increased the proportion of side population (SP) cells and upregulated the expression of the stem cell-associated genes *Nanog*, *OCT4*, and *SOX2*, while its inhibition resulted in opposite effects. Taken together, our findings demonstrated that PIWIL1 induced drug resistance by activating mitophagy and regulating the MM stem cell population. PIWIL1 depletion significantly overcame drug resistance and could be used as a novel therapeutic target for reversing resistance in MM patients.

## Introduction

Multiple myeloma (MM) is the most common plasmacytoma. With the development of new drugs and stem cell transplantation, early treatment in most MM patients has favorable results. However, nearly all patients will eventually relapse, exhibiting multidrug resistance. Therefore, there is an urgent need to explore novel mechanisms and therapeutic strategies to overcome drug resistance.

The PIWI-like RNA-mediated gene silencing 1 (PIWIL1) protein is an important member of the Argonaute protein family, and is highly expressed in germ cells; stem cells; a variety of cancer cells, including seminoma, gastric cancer, breast cancer, lung cancer, and colorectal cancer cells (1–6). Accumulating evidence has revealed that PIWIL1 is implicated in proliferation, apoptosis, invasion, migration, and angiogenesis in a variety of cancers, and it is closely related to poor histological grading of tumor tissue (1, 5, 7). In addition, PIWIL1 is essential for stem cell maintenance and the self-renewal of germ stem cells, hematopoietic stem cells, mesenchymal stem cells, and lung cancer stem cells (CSCs) (2, 3, 8–10). Clinically, higher-level PIWIL1 is positively correlated with advanced clinical stage and worse overall survival of cancer patients (3, 6, 11–15). Recently, PIWIL1 has been demonstrated to be associated with drug resistance. Ectopic PIWIL1 increased the chemical resistance of SiHa cervical cancer cells by regulating stem cell self-renewal-associated transcription factors (16). However, little is known about the role of PIWIL1 in the pathogenesis and drug resistance of MM. Our previous studies have demonstrated that the small PIWI-interacting RNA piRNA-823 plays an important role in myeloma growth, vasculogenesis, and migration (17). Therefore, we speculated that PIWIL1 proteins might also contribute to myelomagenesis and drug resistance in MM, and further investigation is required to elaborate the underlying molecular mechanism.

Autophagy is a lysosome-dependent protein degradation pathway to recycle excessive protein aggregates, including damaged or dysfunctional organelles (18). Most studies have found that autophagy can protect MM cells by eliminating misfolded proteins and promote the occurrence and progression of MM (19–21). Moreover, a growing number of studies have observed a correlation between autophagy and drug resistance (22–24). Inhibition of autophagy can increase the susceptibility of myeloma cells to apoptosis and have a strong anti-MM effect (25, 26). Mitophagy is a novel selective autophagy in which target mitochondria are recognized by autophagosomes and delivered to lysosomes for degradation (27, 28). There is increasing evidence that dysregulation of mitophagy is associated with a broad range of cancers (29–32). Cancer cells can use mitophagy to recycle intracellular components under conditions of metabolic stress or during anticancer treatments and participate in chemotherapy resistance. However, the role of mitophagy in the pathogenesis of MM remains elusive. Several studies have demonstrated that the anti-MM agents bortezomib, adriamycin, and dexamethasone cause cellular toxicity partially by inducing mitochondrial dysfunction and enhancing superoxide formation (33–35). Therefore, autophagy/mitophagy might be a novel target for reversing chemotherapeutic drug resistance in MM.

Here, we demonstrated that PIWIL1 was highly expressed in CD138+ cells derived from patients with newly diagnosed MM, and its high expression was associated with disease stage. PIWIL1 plays a vital role in MM tumor progression and drug resistance by modulating mitophagy and regulating MM stem cells, and PIWIL1 suppression appears to be a promising novel therapeutic strategy for MM.

## Materials and Methods

### Patient Samples and Bone Marrow Plasma Cell Isolation

The study protocol was approved by the Institutional Review Board of Wuhan Union Hospital, Huazhong University of Science and Technology, Wuhan, China, and all study participants provided written informed consent. A total of 20 patients with newly diagnosed MM, 10 cases with relapse or refractory MM, and 5 healthy volunteers who visited our hospital between April 2017 and March 2020 were enrolled in this study. All patients with MM were diagnosed according to and met the International Myeloma Working Group consensus criteria for MM. None of the newly diagnosed patients had received any specific anti-MM therapy before enrollment. CD138^+^ cells were freshly isolated from the bone marrow of MM patients and normal healthy donors, and purified using anti-CD138 microbeads on an AutoMACS system (Miltenyi Biotec, Auburn, CA, USA) according to the manufacturer’s instructions.

### Antibodies and Reagents

The following primary antibodies were used in this study: anti-PIWIL1 antibody (ab12337; Abcam, Cambridge, UK), anti-p-mTOR antibody (ab109268; Abcam), anti-LC3 I/II (#2775S; Cell Signaling Technology, Danvers, MA, USA), anti-P62/SQSTM1 (#8025S; Cell Signaling Technology), anti-Parkin (#4211; Cell Signaling Technology), anti-phosphor-NAK/TBK (ab109272; Abcam), anti-NAK/TBK (A5497; Bimake), anti-optineurin (ab213556; Abcam), anti-β-actin (#4970; Cell Signaling Technology), anti-Akt (#4691; Cell Signaling Technology), anti-phospho-Akt (Ser473) (#9271; Cell Signaling Technology), anti-Ki67 (#4685; Cell Signaling Technology), anti-Bcl-2 (A5010; Bimake), anti-OCT4 (60242-1-Ig; Proteintech), and anti-Nanog (#4903T; Cell Signaling Technology). Goat anti-mouse and goat anti-rabbit antibodies (Biosciences) were used as the secondary antibodies for western blotting. Dexamethasone and doxorubicin were purchased from Sigma (St. Louis, MO, USA), and bortezomib was purchased from Selleck Chemicals. 3-Methyladenine (3-MA) and the mitophagy inhibitor cyclosporin A (CsA) were purchased from MedChemExpress (NJ, USA). All were dissolved in dimethyl sulfoxide (DMSO) and stored at −20°C for up to 6 months. For all cell-based experiments, drugs were diluted at least by 1:1000 to ensure that the final DMSO concentration was lower than 0.1%.

### Cell Culture

Human RPMI-8226, NCL-H929, U266, ARH77, MM1R, and MM1S cell lines were obtained from the Chinese Academy of Sciences Committee Type Culture Collection (Shanghai, China) and cultured at 37°C in a humidified atmosphere containing 5% CO_2_ in RPMI-1640 medium supplemented with 10% fetal bovine serum (Gibco), 100 units/mL penicillin, and 100 μg/mL streptomycin.

### Total RNA Extraction and qRT-PCR

Total RNA was extracted from cells and reverse transcribed with a Prime Script RT reagent kit from TaKaRa (Otsu, Japan) according to the manufacturer’s instructions. Expression levels of the target genes were then analyzed by qPCR using a SYBR^®^ Premix Ex-Taq™ II Kit from Bio-Rad (Ca, USA). The housekeeping gene *β-actin* was used as an internal control. Primers were synthesized by Tsingke (Beijing, China). The primers used for the qRT-PCR (primer sequence 5′–3′) included PIWIL1, GTCTGTTGTCAAGTAATCGGAAGG (forward) and TTGCTGTTTGCCTAAGGTTCG (reverse); β-actin, CTCCATCCTGGCCTCGCTGT (forward) and GCTGTCACCTTCACCGTTCC (reverse); OCT4, GGTCCGAGTGTGGTTCTGTA (forward) and GCAGCCTCAAAATCCTCTCG (reverse); NANOG, TAATAACCTTGGCTGCCGTCT (forward) and GCCTCCCAATCCCAAACAATA (reverse); SOX2, GGGGTGCAAAAGAGGAGAGTA (forward) and TGTCATTTGCTGTGGGTGATG (reverse).

All RT-PCR analyses were performed in triplicate, and the results were analyzed by the 2^–ΔΔCt^ method.

### Western Blot

For western blot analysis, whole-cell lysates were prepared using RIPA buffer (Thermo Fisher Scientific) in the presence of a protease inhibitor and PhosStop (Roche, Basel, Switzerland). The protein concentration was measured using a bicinchoninic acid (BCA) Kit (Thermo Fisher Scientific). Briefly, 30 μg protein samples were separated and transferred onto polyvinylidene fluoride (PVDF) membranes. The membranes were then blocked and incubated with primary antibodies at 4°C overnight, then washed and probed by incubating them with the appropriate secondary antibodies for 60 min at room temperature. After the final wash, the membranes were visualized using enhanced chemiluminescence detection reagent (ECL; Thermo Fisher Scientific). As a measure of protein levels, band density was analyzed using ImageJ software (National Institutes of Health). For densitometry, the protein level of the NC group was arbitrarily set as 1(100%) in each blot. Protein levels were determined as the ratio to the levels of NC groups and normalized to the levels of loading controls. The loading controls of phospho-proteins are total proteins. Others are normalized to β-actin levels. LC3 levels are display by LC3A/B ratio and normalized to β-actin.

### Cell Transfection

A lentivirus-containing green fluorescence protein with a short hairpin RNA against human PIWIL1, an overexpression lentiviral vector, and a control lentivirus were obtained from GeneChem Biotechnology (Shanghai, China), transfected into MM cells according to the manufacturer’s instructions, and screened with 3 µg/mL puromycin (Sigma) to acquire pooled cell populations.

The interference sequence against PIWIL1 and the control sequence were TGTGAGAAGTGGTAGTGTT and TTCTCCGAACGTGTCACGT, respectively (5ʹ–3ʹ for both).

The PIWIL1 overexpression sequence and control sequence were GTTTGGTTTGTTGGGTTTGTG and TTCTCCGAACGTGTCACGT, respectively (5ʹ–3ʹ for both).

### Cell Counting Kit-8 Assay

Cell proliferation was determined using a Cell Counting Kit-8 (CCK-8) (MedChemExpress, NJ, USA) assay in accordance with the manufacturer’s instructions. Briefly, transfected RPMI-8226 and NCL-H929 cells were plated in 96-well plates at a density of 8 × 10^3^ cells/well. Chemotherapy drugs, including bortezomib, dexamethasone, and doxorubicin, were added to the plates when needed. After 48 h, 10 μL of CCK-8 solution was added to each well and further incubated for 1 h. Absorbance was measured at a wavelength of 450 nm using a Bio-Rad microplate reader. Cell viability was calculated as optical density (OD) of the treated groups/OD values of the control groups × 100%.

### Transmission Electron Microscopy

Stably transfected RPMI-8226 and NCL-H929 cells were centrifuged at 1000 rpm for 5 min and fixed overnight in electron microscopy fixative (4% glutaraldehyde and 1% osmic acid buffer). Cells were embedded in epoxy resin, prepared into semithin sections for localization, and then cut into ultrathin sections. The cells were stained with uranyl acetate and lead citrate and then observed under a transmission electron microscope (JEM-2000EX; JEOL Ltd., Tokyo, Japan).

### Flow Cytometry

#### Apoptosis Fluorometric Assay

A sample of transfected MM cells (1 × 10^6^ cell/mL) was harvested, washed twice with phosphate-buffered saline (PBS), and incubated with 5 μL of annexin V-PE and 5 μL of 7-AAD (BD Biosciences, San Jose, Ca, USA) for 15 min at room temperature in the dark. Then, 1× Binding Buffer (400 μL) was added to each tube, and the samples were analyzed by flow cytometry within 1 h.

#### SP Cell Detection

A sample of transfected MM cells (1 × 10^6^ cell/mL) was harvested, washed twice with PBS, incubated with 5 µg/mL Hoechst 33342 (Sigma) for 90 min at 37°C, and analyzed using a flow cytometer. Verapamil (Sigma) was used in the control group.

#### Mitochondrial Calcium Measurements

A sample of transfected MM cells (1 × 10^6^ cell/mL) was harvested, washed with PBS, and incubated with 10 μM Rhod-2/AM (Invitrogen) for 30 min at 37°C in the dark. After washing the cells twice with PBS, the stained cells were examined by flow cytometry.

#### Mitochondrial Reactive Oxygen Species Accumulation Assay

A sample of transfected MM cells (1 × 10^6^ cell/mL) was harvested and washed twice with PBS, and the levels of mitochondrial ROS were assessed using 5 µM of Mito-SOX Red (Invitrogen) according to the manufacturer’s instructions, and analyzed by flow cytometry.

### Human Tumor Xenografts in NOD/SCID Mice

Four-week-old male NOD/SCID mice were purchased from Vital River Laboratory Animal Technology Co., Ltd. (Beijing, China). Then, 5 × 10^6^ transfected RPMI-8226 cells and NCL-H929 cells were subcutaneously injected into the right flanks of the mice (n = 5 for each group). Tumor growth was monitored every 3 days. After 4 weeks, the mice were sacrificed, and the tumors were harvested. For the chemotherapy study, 5 × 10^6^ transfected PIWIL1^+^ 8226 cells and negative control cells were subcutaneously injected into the right flank of the mice. After 2 weeks, the mice were randomly divided into three groups and intraperitoneally injected with either 3-MA (3 mg/kg, twice weekly), bortezomib (0.5 mg/kg, twice weekly), or 3-MA plus bortezomib for three weeks. Mice were sacrificed at 42 days, and tumors were collected for assessment. The tumor diameter was measured every 3 days, and the tumor volume was calculated as (a^2^ × b)/2, where a is the width and b is the length. All the experiments were performed in accordance with the procedures of the Animal Research and Care Committee of Huazhong University of Science and Technology, Wuhan, China.

### Immunohistochemical Staining

Paraffin-embedded sections of tumor tissues from xenograft mice were incubated with antibodies against p-mTOR, Ki67, Nanog, OCT4, optineurin, and p62 at 4°C overnight. Subsequently, the sections were treated with polymer, developed with a diaminobenzidine (DAB) chromogen, and counterstained with hematoxylin.

### Statistical Analysis

All data are presented as the means ± standard error of the mean (SEM). The mean values were calculated on the basis of data obtained from at least three independent experiments conducted on separate days using freshly prepared reagents. A two-tailed Student’s *t* test was used to determine significant differences between the two groups. All the analyses were performed using GraphPad Prism 8.0 (GraphPad Software, Ca, USA). All p values <0.05 were considered statistically significant.

## Results

### PIWIL1 Expression Is Increased in MM Cell Lines and Primary MM Cells

**Figure 1:**
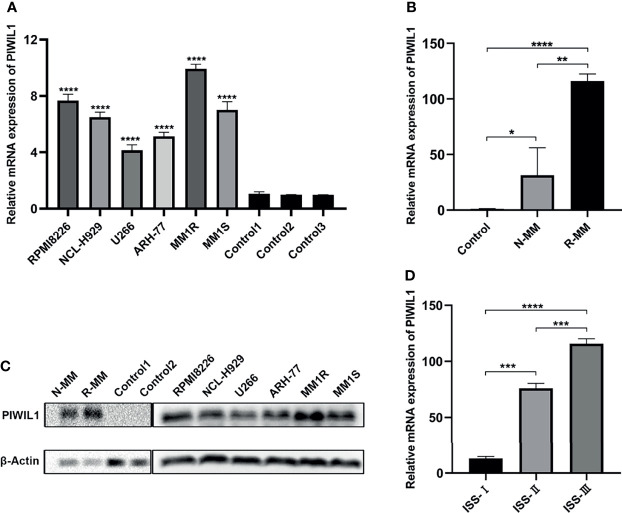
PIWIL1 is highly expressed in MM cells, and its expression is related to disease stage. **(A, B)** Quantitative reverse-transcription polymerase chain reaction (qRT-PCR) results of PIWIL1 expression in MM cell lines and primary MM cells. **(C)** Results of the western blot analysis to detect PIWIL1 expression in MM cell lines, primary MM cells, and healthy donors (control). **(D)** qRT-PCR results of PIWIL1 expression in different disease stages of primary MM cells. ISS I (n = 2), ISS II (n = 6), and ISS III (n = 12), Control: healthy donors (n = 5), N-MM, newly diagnosed MM patients (n = 20), and R-MM, relapsed or refractory MM patients (n = 10). Data are from representative images are expressed as the means ± SEM of three independent experiments (*P < 0.05, **P < 0.01, ***P < 0.001, ****P < 0.0001).

The results of the qRT-PCR revealed significantly higher levels of PIWIL1 transcript in MM cell lines than in normal bone marrow (BM) samples (n = 5; **Figure 1A**). To establish the translational relevance of *in vitro* data, we measured the *PIWIL1* mRNA expression levels in BM-derived CD138^+^ cells in MM patients, including 20 patients with newly diagnosed MM (N-MM) and 10 patients with relapsed or refractory MM (R-MM). *PIWIL1* mRNA expression was found to be higher in N-MM BM samples than in normal BM samples (n = 5; **Figure 1B**). In addition, R-MM patients expressed higher *PIWIL1* levels than N-MM patients, suggesting a progressive increase in *PIWIL1* expression from patients with newly diagnosed MM to those with relapsed/refractory MM. Similarly, MM cell lines and MM primary cells exhibited higher levels of PIWIL1 protein expression than the normal control, as determined by western blot analysis (**Figure 1C**). Further subgroup analysis indicated that PIWIL1 expression was remarkably higher in stage II and stage III MM patients than in stage I MM patients (International Staging System [ISS] stage; **Figure 1D**), indicating that high PIWIL1 levels are associated with advanced disease stage.

### PIWIL1 Promotes proliferation and Mediates Drug Resistance in MM Cells

**Figure 2:**
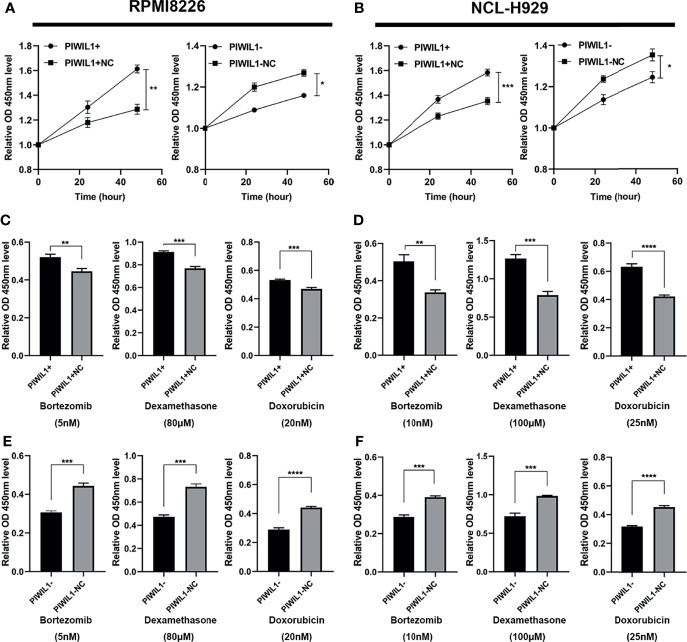
PIWIL1 promotes proliferation and mediates drug resistance in MM cells. **(A, B)** Cell viability of different groups of transfected RPMI-8226 cells **(A)** and NCL-H929 cells **(B)** measured using the CCK-8 assay. **(C–F)** Different groups of transfected MM cells were treated with dexamethasone, bortezomib, and doxorubicin at the indicated concentrations. After 48 h, cell viability was measured using the CCK-8 assay. Data are expressed as the mean ± SEM of each group from three separate experiments (*P < 0.05, **P < 0.01, ***P < 0.001, ****P < 0.0001).

To investigate whether PIWIL1 promotes MM cell growth to acquire malignant/progressive traits, we used lentiviral transfection to stably establish the PIWIL1-overexpressing MM cell lines PIWIL1^+^ 8226 and PIWIL1^+^ 929; the PIWIL1-downregulated MM cell lines PIWIL1^–^ 8226 and PIWIL1^–^ 929; and their negative controls, PIWIL1^+^ 8226 NC, PIWIL1^+^ 929 NC, PIWIL1^–^ 8226 NC, and PIWIL1^–^ 929 NC. **Supplementary Figure 1** shows the efficiency of transfection. Results of the CCK-8 assay showed that PIWIL1 downregulation caused a significant decrease in cell viability, and PIWIL1 overexpression resulted in higher cell viability compared with that in the NC group (**Figures 2A, B**), suggesting that PIWIL1 promotes the proliferation of MM.

To investigate whether PIWIL1 mediated chemoresistance in MM, we exposed a panel of transfected RPMI-8226 MM and NCL-H929 cells to the indicated concentrations of bortezomib (5 nM and 10 nM, respectively), dexamethasone (80 μM and 100 μM, respectively), or doxorubicin (20 nM and 25 nM, respectively). Subsequent CCK-8 assays revealed that PIWIL1 overexpression resulted in the resistance of MM cells to bortezomib, dexamethasone, and doxorubicin, while PIWIL1 downregulation induced re-sensitization of MM cells to chemotherapy (**Figures 2C**–**F**). Taken together, these results illustrated that PIWIL1 mediated the drug resistance of MM *in vitro*.

### PIWIL1 Fosters Drug Resistance Through Autophagy and Mitophagy Instead of Through Apoptosis

The role of PIWIL1 in apoptosis induction in glioblastoma is well known (36). Therefore, we investigated the impact of PIWIL1 on MM cell apoptosis. Interestingly, the apoptosis rates or the expression of the apoptosis-related proteins Bcl-2 and cleaved caspase-3 did not differ significantly between compared groups (**Supplementary Figure 2**).

**Figure 3:**
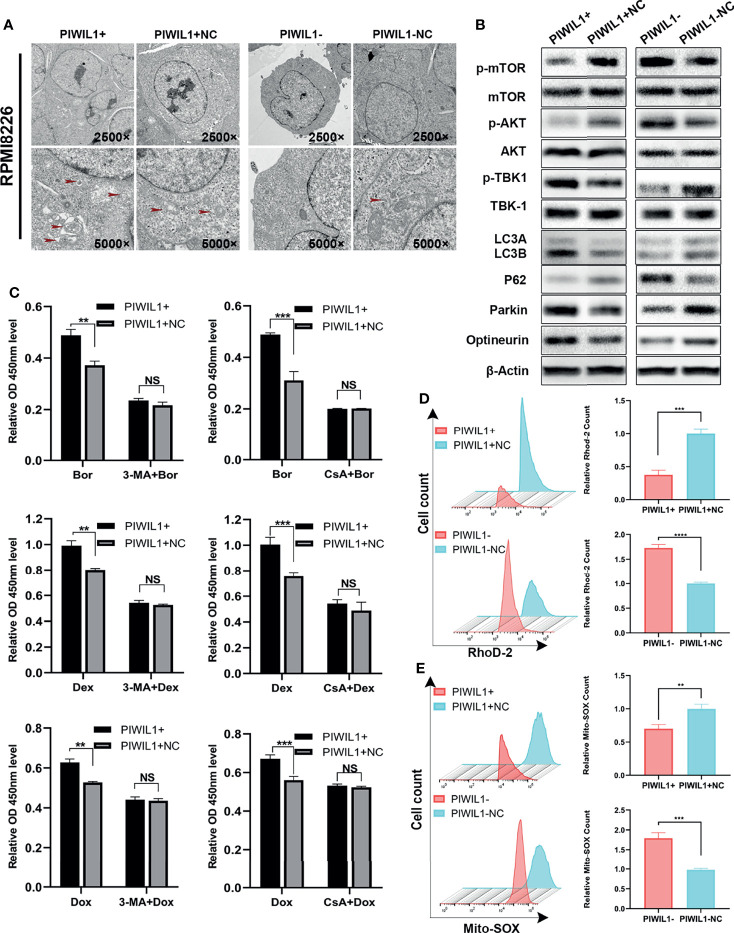
PIWIL1 fosters drug resistance through autophagy and mitophagy in RPMI-8226 cells. **(A)** Transmission electron microscopy of the morphological characteristics of MM cells after transfection. Red arrows indicate autophagosomes or autolysosomes. Panel shows the double-layered membrane engulfing a mitochondrion (early stage of mitophagy) and the double-layered membrane containing a degraded mitochondrion (late stage of mitophagy). **(B)** A representative result of the autophagy- and mitophagy-related protein levels detected by Western blot. **(C)** Different groups of transfected RPMI-8226 MM cells were pretreated with the same dose of solvent, autophagy inhibitor 3-MA (5 mM) for 12 h or mitophagy inhibitor CsA (5 μM) for 24 h, followed by dexamethasone (80 μM), bortezomib (5 nM), and doxorubicin (20 nM) for 48 h. The cell viability of the different groups of transfected MM cells was assessed using the CCK-8 assay. **(D)** The left panel shows representative images of mitochondrial calcium changes in the different groups. The right panel shows the mitochondrial calcium level presented as the relative mean fluorescent intensity (MFI) of Rhod-2 staining in RPMI-8226 cells. **(E)** The left panel shows representative images of changes in the mitochondrial ROS level in different groups. The right panel shows mitochondrial ROS levels presented as the relative mean fluorescent intensity (MFI) visualized following Mito-SOX staining of RPMI-8226 cells. Data are from representative images or are expressed as the means ± SEM of each group from three separate experiments (**P < 0.01, ***P < 0.001, **** P < 0.0001, NS, p> 0.05).

Therefore, we assumed that PIWIL1 modulated other cell death mechanisms in MM. Transmission electron microscopy showed that the number of autophagosomes with double-membrane structures containing swollen and dilated mitochondria was decreased in PIWIL1-downregulated MM cells. Conversely, the autophagic and mitophagic responses in PIWIL1-overexpressing MM cells were stronger than those in NC cells (**Figure 3A** and **Supplementary Figure 3A**).

We then searched for the autophagy- and mitophagy-related proteins regulated by PIWIL1. As shown in **Figure 3B** and **Supplementary Figures 3B**, **6A, C**, we observed a significant increase in the LC3B and a decrease in P62, which are autophagy activity monitors, in the PIWIL1-overexpressing group compared to the NC group. Furthermore, the phosphorylation levels of both the mTOR and AKT-Ser473 (a substrate of the mTORC2 complex) were decreased in PIWIL1-overexpressing MM cells compared with NC cells. In addition, the expression levels of the canonical PINK1/Parkin pathway proteins Parkin, optineurin, and p-TBK-1 were significantly increased in PIWIL1-overexpressing MM cells compared with NC cells. The opposite results were found in the PIWIL1-downregulated group (**Figure 3B** and **Supplementary Figures 3B**, **6B, D**).

Calcium is known to be a key regulator of mitochondrial function (37). As shown in **Figure 3D** and **Supplementary Figure 3D**, mitochondrial calcium was decreased in PIWIL1-overexpressing MM cells relative to that in NC cells, and PIWIL1-downregulated MM cells significantly accumulated mitochondrial calcium. Mitochondrial calcium overload has been shown to promote ROS generation in mitochondria (38). Flow cytometry analysis showed that the Mito-SOX intensity was decreased in PIWIL1-overexpressing MM cells compared with that in NC cells, and increased in PIWIL1-downregulated cells compared with that in NC cells (**Figure 3E** and **Supplementary Figure 3E**).

To substantiate whether PIWIL1 induced drug resistance through autophagy/mitophagy, we treated transfected MM cells with 5 mM of the autophagy inhibitor 3-methyladenine (3-MA) for 12 h or 5 µM of the mitophagy inhibitor cyclosporin A (CsA) for 24 h. These pretreated cells were then exposed to dexamethasone, bortezomib, or doxorubicin for 48 h. Results of the CCK-8 assay analysis revealed no significant difference in cell viability between the PIWIL1-overexpressing group and the NC group (**Figure 3C** and **Supplementary Figure 3C**). Together, these results suggested that PIWIL1 serves as a key regulator of mitochondrial function and induces drug resistance through autophagy and mitophagy in MM.

### PIWIL1 Regulates the Stemness of MM Cells

**Figure 4:**
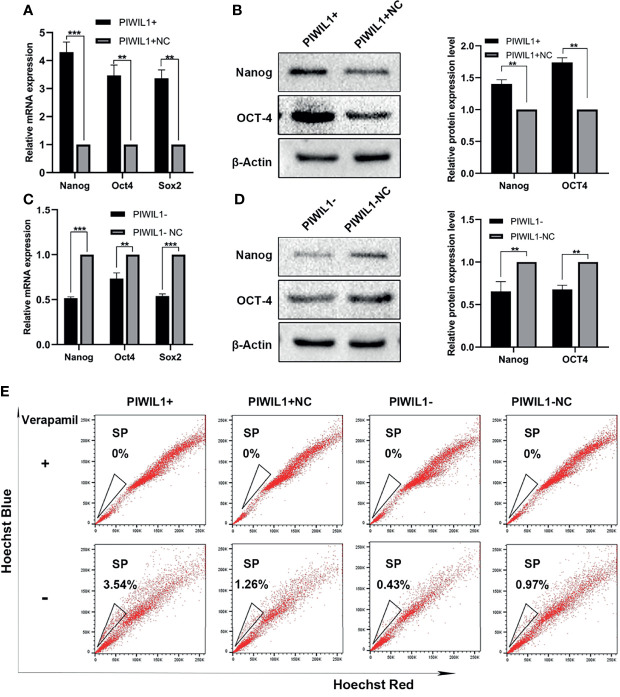
PIWIL1 regulates the stemness of RPMI-8226 MM cells. **(A, C)** qRT-PCR results of the expression of stemness-related genes such as Nanog, SOX2, and *OCT4* in different groups of transfected MM cells. **(B, D)** Left panel: Western blot results of the expression of stemness-related proteins such as Nanog and OCT4 in different groups of transfected MM cells; right panel: Histograms show the quantification of immunoblot images of Nanog and OCT4 proteins in MM cell as measured by ImageJ software. **(E)** Representative flow cytometric analysis of Hoechst-33342-based SP staining in transfected RPMI-8226 cells. Data are from representative images or are expressed as the mean ± SEM of each group from three separate experiments (**P < 0.01, ***P < 0.001).

Several studies have demonstrated that PIWIL1 is essential for stem cell maintenance and self-renewal of CSCs (7), which is associated with drug resistance. The SP cell population has been widely regarded as the CSCs for MM. PIWIL1 downregulation significantly depleted SP cells (**Figure 4** and **Supplementary Figure 4**). In addition, results of the qRT-PCR and western blot analyses revealed that the expression levels of the stemness-related genes *Nanog*, *SOX2*, and *OCT4* were significantly reduced in PIWIL1-downregulated MM cells. The converse was seen in the case of PIWIL1-overexpressing MM cells, suggesting that PIWIL1 regulates the stemness of MM cells.

### PIWIL1 Promotes the Growth of Xenograft Tumors and Attenuates Their Sensitivity to Chemotherapy *In Vivo*

**Figure 5:**
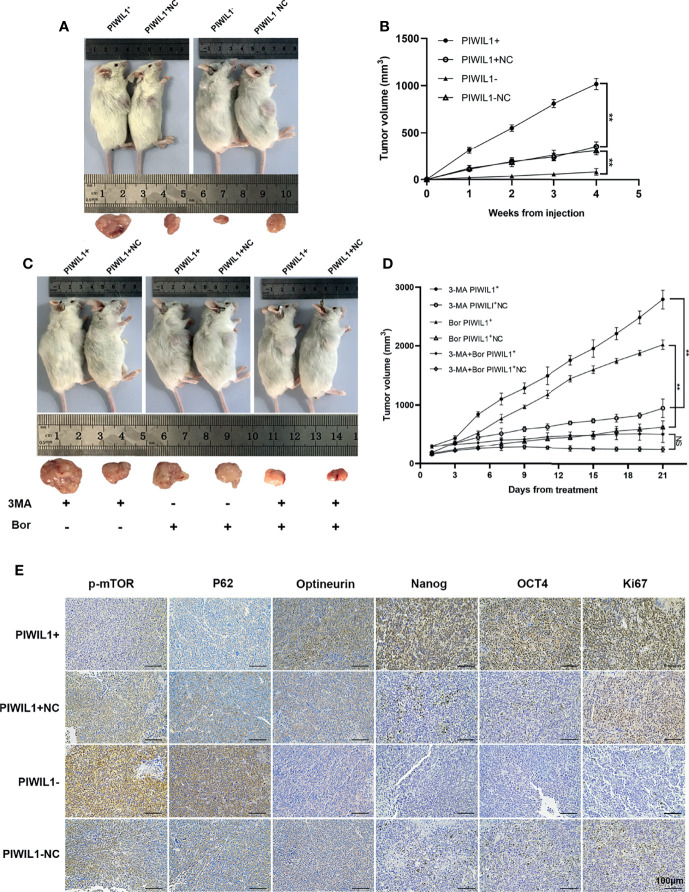
PIWIL1 promotes the growth of xenograft tumors and enhances resistance of MM cells to chemotherapy *in vivo*. **(A)** Tumor volume of NOD/SCID mice xenografted with different groups of transfected RPMI-8226 cells. **(B)** Tumor growth curves of NOD/SCID mice after transfected RPMI-8226 cell injection. **(C, D)** Tumor volume and tumor growth curves of xenograft tumors after bortezomib treatment. **(E)** Representative immunohistochemical staining of p-mTOR, P62, optineurin, Nanog, OCT4, and Ki67 in tumor xenografts. Data are from representative images of three separate experiments. (**P < 0.01, NS, p > 0.05).

To evaluate the role of PIWIL1 in MM cell proliferation *in vivo*, human tumor xenograft mice were established as described in Materials and Methods. The tumor burden was significantly higher in PIWIL1^+^ mice than in control mice and significantly lower in PIWIL1^–^ mice (**Figures 5A, B**). In accordance with this finding, results of the immunohistochemical analyses revealed increased Ki67 expression, decreased expression of autophagy-related proteins (p-mTOR and p62), and increased expression of the mitophagy-related protein optineurin and stem cell-related proteins (OCT4 and Nanog) in the PIWIL1^+^group, illustrating that PIWIL1 promoted cell proliferation through mitophagy and the stem cell population *in vivo* (**Figure 5E**). The opposite results were observed in the PIWIL1-downregulated group (**Figures 5A, B, E**). Similar results were observed in xenograft tumor models established with transfected NCL-H929 cells (**Supplementary Figure 5**).

To validate the role of autophagy in PIWIL1-mediated drug resistance *in vivo*, mice xenografted with PIWIL1^+^ 8226 MM cells and NC MM cells were intraperitoneally injected with 3-MA, bortezomib, or a combination of the two. The results showed that the tumor mass was significantly higher in mice treated with bortezomib monotherapy and xenografted with PIWIL1^+^ 8226 MM cells than in the NC group, whereas the tumor burden was significantly lower in PIWIL1^+^ 8226 MM mice treated with 3-MA plus bortezomib than in mice treated with bortezomib monotherapy (**Figures 5C, D**).

## Discussion

Despite the development of novel therapeutic strategies for multiple myeloma (39), chemoresistance inevitably develops and contributes to poor patient outcomes. Thus, to understand the reason for this therapeutic failure, drug resistance mechanisms must be characterized, and it is imperative to identify novel targets to reverse chemoresistance and to improve the success of chemotherapy.

The PIWI protein family, which is highly conserved across species (3), has emerged as an attractive target for cancer treatment due to its restrictive expression and function in tumorigenesis. The PIWIL1 protein has been found to be specifically expressed or dysregulated in many tumors and its expression is correlated with poor clinical outcomes (7, 40). Our data first revealed increased PIWIL1 levels in MM patients and MM cell lines, and high PIWIL1 expression was linked to advanced disease stage and refractoriness/relapse, suggesting that aberrantly expressed PIWIL1 can be employed as a new class of diagnostic and prognostic biomarker during MM development. In addition, PIWIL1 overexpression significantly promoted the proliferation of MM cells. PIWIL1 downregulation inhibited myeloma growth in xenograft models, and restraining the PIWIL1-regulated signaling pathway sensitized MM to the chemotherapeutic agents bortezomib, doxorubicin, and dexamethasone *in vitro* and *in vivo*, indicating that PIWIL1 is a potential therapeutic target for MM patients.

CSCs are the origin of cancer chemoresistance and recurrence and have been recognized as crucial tumor-initiating cells. These relapse-initiating cells, which persist after chemotherapy, represent potential targets to prevent drug resistance and relapse. As a CSCs marker, PIWIL1 has been found to promote the proliferation of glioblastoma CSCs and regulate their self-renewal and differentiation (2, 41). Ectopic PIWIL1 overexpression in cervical cancer cells promotes tumor sphere formation by regulating the stem cell-related transcription factors *OCT4*, *NANOG*, *KLF4*, and *BMI1*, thus conferring resistance to cisplatin (16). In the present study, we demonstrated for the first time that PIWIL1 is more highly expressed in R-MM patients than in N-MM patients and that PIWIL1 overexpression significantly confers chemoresistance to MM cells. PIWIL1 inhibition facilitates the sensitivity of MM cells to doxorubicin, bortezomib, and dexamethasone by decreasing the MM stem cell population and regulating stem cell pluripotency genes, including *NANOG*, *OCT4*, and *SOX2*. These data determine the role of PIWIL1 as a pan-resistance target, and PIWIL1 could serve as a novel therapeutic target for reversing chemoresistance in patients with refractory/relapsed MM.

Most studies found that PIWIL1 knockdown suppresses tumor cell and stem cell growth by regulating cell cycle arrest, senescence, or apoptosis (40–42). However, here we observed no change in the apoptosis rate after PIWIL1 upregulation and downregulation. Instead, our results revealed that PWIL1 mainly affects another important cell death mechanism, namely, autophagy/mitophagy in MM cells. Recently, mitochondria have emerged as signaling entities involved in the self-renewal, commitment, and differentiation of stem cell functions. Mitophagy, as an essential mitochondrial quality control mechanism, is beginning to attract more attention in the field of stem cell research, and it has been reported to play a vital role in the maintenance and stress response of hematopoietic stem cells (43). Furthermore, mitophagy has been shown to contribute to tumorigenesis and is essential for the maintenance of hepatic cancer stem cells (44). In the present study, PIWIL1 was found to modulate autophagy-related proteins LC3, p62, and mTOR and the canonical PINK1/PARKIN pathway proteins, indicating that PIWIL1 promoted myeloma growth by modulating autophagy/mitophagy. An *in vivo* gain-of-function study demonstrated that hyperactivation of PINK1/PARKIN-dependent mitophagy caused a significant reduction in differentiated blood cells with a concomitant expansion of the hematopoietic stem cell pool (45). A detailed mechanism revealed by Xu et al. was that p62/SQSTM1 improves breast cancer stem-like properties by promoting MYC mRNA stabilization (46). Hence, the stemness of MM cells might be related with mitophagy modulated by PIWIL1 regardless of Parkin-dependent or not. In addition, low ROS levels are known to preserve quiescence and self-renewing capacity. Mitophagy prevents the generation of reactive oxygen species (ROS) to achieve the bioenergetics need of CSCs upon various metabolic and extracellular stress (47, 48). Here, we revealed that PIWIL1-overexpressing MM cells exhibited an increase in the number of mitophagosomes and mitochondrial with low mitochondrial ROS and mitochondrial calcium levels. Therefore, our data positively correlate PIWIL1 with mitophagy, indirectly supporting the critical role of mitophagy in regulating CSCs.

Mitophagy in CSCs has been determined to be a major contributor to the drug resistance of tumors. Silencing the mitophagy regulator BNIP3L has been shown to significantly halt mitophagy and as a result enhance sensitivity of colorectal CSCs to doxorubicin (49). Like autophagy, mitophagy is believed to play a dual role in cancer drug resistance depending on the level of autophagy and the different types of tumors and stages of tumor progression (50). In some studies, mitophagy increases cancer cell sensitivity to chemotherapy by maintaining cellular homeostasis and preventing oncogenic transformation (51). In contrast, mitophagy promotes cancer cell survival and confers cell resistance to chemotherapeutic stress by degrading damaged mitochondria and reducing mitochondrial reactive oxygen species (52). In our research, a mitophagy inhibitor significantly improved the sensitivity of PIWIL1-overexpressing MM cells to chemotherapeutic drugs, validating the role of mitophagy in inducing MM chemoresistance. In summary, here we identified a novel functional mechanism by which PIWIL1 confers chemotherapeutic drug resistance by activating mitophagy and the myeloma stem cell population through AKT-mTOR and PARKIN-dependent mitophagy pathways. Nevertheless, the exact molecular target of PIWIL1 in regulating these phenotypes remain to be elucidated. From the Protein-Protein interaction networks functional enrichment analysis through Online tools STRING and GeneMANIA, we figured out that SND1 and MAEL might be the target gene of PIWIL1 (53, 54). The oncogene role of MAEL and SND1 have been shown to enhance the development and maintain the stemness of carcinoma (55–61). Furthermore, SND1 promoted the proliferation of cancer cells and regulated autophagy by activating NF−κB (62–67). Further research should be undertaken to investigate more downstream molecular target regulated by PIWIL1 relating to mitophagy and myeloma stem cells. These can facilitate the development of novel strategies for the treatment of MM.

## Data Availability Statement

The original contributions presented in the study are included in the article/**Supplementary Material**. Further inquiries can be directed to the corresponding author.

## Ethics Statement

The studies involving human participants were reviewed and approved by institutional review board of Wuhan Union Hospital, Huazhong University of Science and Technology, Wuhan, China. The patients/participants provided their written informed consent to participate in this study. The animal study was reviewed and approved by Animal Research and Care Committee of Huazhong University of Science and Technology, Wuhan, China

## Author Contributions

QW conceived the studies. YW and QW designed the studies. YW and LY performed the experiments. YW and YT wrote the manuscript. YW and HY prepared the stables and figures. All authors contributed to the article and approved the submitted version.

## Funding

The correlative laboratory studies were funded by the National Natural Science Foundation of P.R. China (no. 81570193, to QW) and no. 81770219 to QW).

## Conflict of Interest

The authors declare that the research was conducted in the absence of any commercial or financial relationships that could be construed as a potential conflict of interest.

## Publisher’s Note

All claims expressed in this article are solely those of the authors and do not necessarily represent those of their affiliated organizations, or those of the publisher, the editors and the reviewers. Any product that may be evaluated in this article, or claim that may be made by its manufacturer, is not guaranteed or endorsed by the publisher.

## Supplementary Material

Supplementary Figure 1 Lentivirus transfection of RPMI-8226 and NCL-H929 cells. **(A, B)** MM cells (RPMI-8226 and NCL-H929) were transfected with a PIWIL1 or a shPIWIL1 lentivirus, and the transfection efficiency was assessed with a fluorescence microscope. **(C, F)** qRT-PCR **(C, D)** and western blot analysis **(E, F)** were used to verify the upregulation or downregulation of PIWIL1 in the lentivirus-transfected cells (n = 3). Data are from representative images or expressed as the means ± SEM of each group from three separate experiments (**** P < 0.0001).

Supplementary Figure 2 PIWIL1 does not regulate apoptosis in MM cells. **(A, C)** Representative flow cytometry results showing apoptosis of MM cells (RPMI-8226 and NCL-H929) after PIWIL1 upregulation or downregulation. **(B, D)** Representative western blot images showing the expression of c-caspase-3 and BCL-2 after PIWIL1 upregulation or downregulation. Data are from representative images of each group from three separate experiments.

Supplementary Figure 3 PIWIL1 fosters drug resistance through autophagy and mitophagy in NCL-H929 cells. **(A)** Transmission electron microscopy of the morphological characteristics of NCL-H929 MM cells after transfection. Arrows indicate autophagosomes or autolysosomes. The panel shows the double-layered membrane engulfing a mitochondrion (early stage of mitophagy) and the double-layered membrane containing a degraded mitochondrion (late stage of mitophagy). **(B)** A representative result of the autophagy- and mitophagy-related protein levels detected by Western blot. **(C)** Different groups of transfected NCL-H929 MM cells were pretreated with the same dose of solvent, autophagy inhibitor 3-MA (5 mM) for 12 h or mitophagy inhibitor CsA (5 μM) for 24 h, followed by dexamethasone (100 μM), bortezomib (10 nM), and doxorubicin (25 nM) for 48 h. Cell viability of the different groups of transfected MM cells was assessed using the CCK-8 assay. **(D)** The left panel shows representative images of mitochondrial calcium changes in different groups. The right panel shows the mitochondrial calcium level presented as the relative MFI of Rhod-2 staining in NCL-H929 cells. **(E)** The left panel shows representative images of mitochondrial ROS level changes in different groups. The right panel shows mitochondrial ROS levels presented as the relative MFI of Mito-SOX staining in NCL-H929 cells. Data are from representative images or are expressed as the means ± SEM of each group from three separate experiments (**P < 0.01, NS, p > 0.05).

Supplementary Figure 4 PIWIL1 can regulate the stemness of NCL-H929 MM cells. **(A)** qRT-PCR results of the expression of stemness-related genes such as *Nanog*, *SOX2*, and *OCT4* in different groups of transfected NCL-H929 MM cells. **(B, D)** Left panel: Western blot results of the expression of stemness-related proteins such as Nanog and OCT4 in different groups of transfected NCL-H929 MM cells; right panel: Histograms show the quantification of immunoblot images of Nanog and OCT4 proteins in MM cell as measured by ImageJ software. € Representative flow cytometric analysis of Hoechst-33342-based SP staining in transfected NCL-H929 cells. Data are from representative images or are expressed as the mean ± SEM of each group from three separate experiments (**P < 0.01, ***P < 0.001).

Supplementary Figure 5 PIWIL1 promotes the growth of xenograft tumors *in vivo*. **(A)** Tumor volume of NOD/SCID mice xenografted with NCL-H929 cells. **(B)** Tumor growth curves of NOD/SCID mice after injection with transfected NCL-H929 cells. **(C)** Representative immunohistochemical staining of p-mTOR, P62, optineurin, Nanog, OCT4, and Ki67 in tumor xenografts. Data are from representative images of three separate experiments (*P < 0.05, **P < 0.01).

Supplementary Figure 6 Relative expression levels of autophagy-related proteins. **(A, B)** Histograms show the quantification of immunoblot images of autophagy-related proteins in transfected RPMI-8226 cells. **(C, D)** Histograms show the quantification of immunoblot images of autophagy-related proteins in transfected NCL-H929 cells. Data are from representative images or are expressed as the means ± SEM of each group from three separate experiments (*P < 0.05, **P < 0.01).

## Abbreviations

MM, multiple myeloma; PIWIL1, PIWI-like protein 1; 3-MA, 3-methyladenine; CsA, mitophagy inhibitor cyclosporin A; SP, side population; CSCs, cancer stem cells.
