# Supplementary figures and images for: PIWIL1 Drives Chemoresistance in Multiple Myeloma by Modulating Mitophagy and the Myeloma Stem Cell Population
Source: Front Oncol. 2022 Jan 10;11:783583. doi: 10.3389/fonc.2021.783583 (PMC8784391; doi:10.3389/fonc.2021.783583)

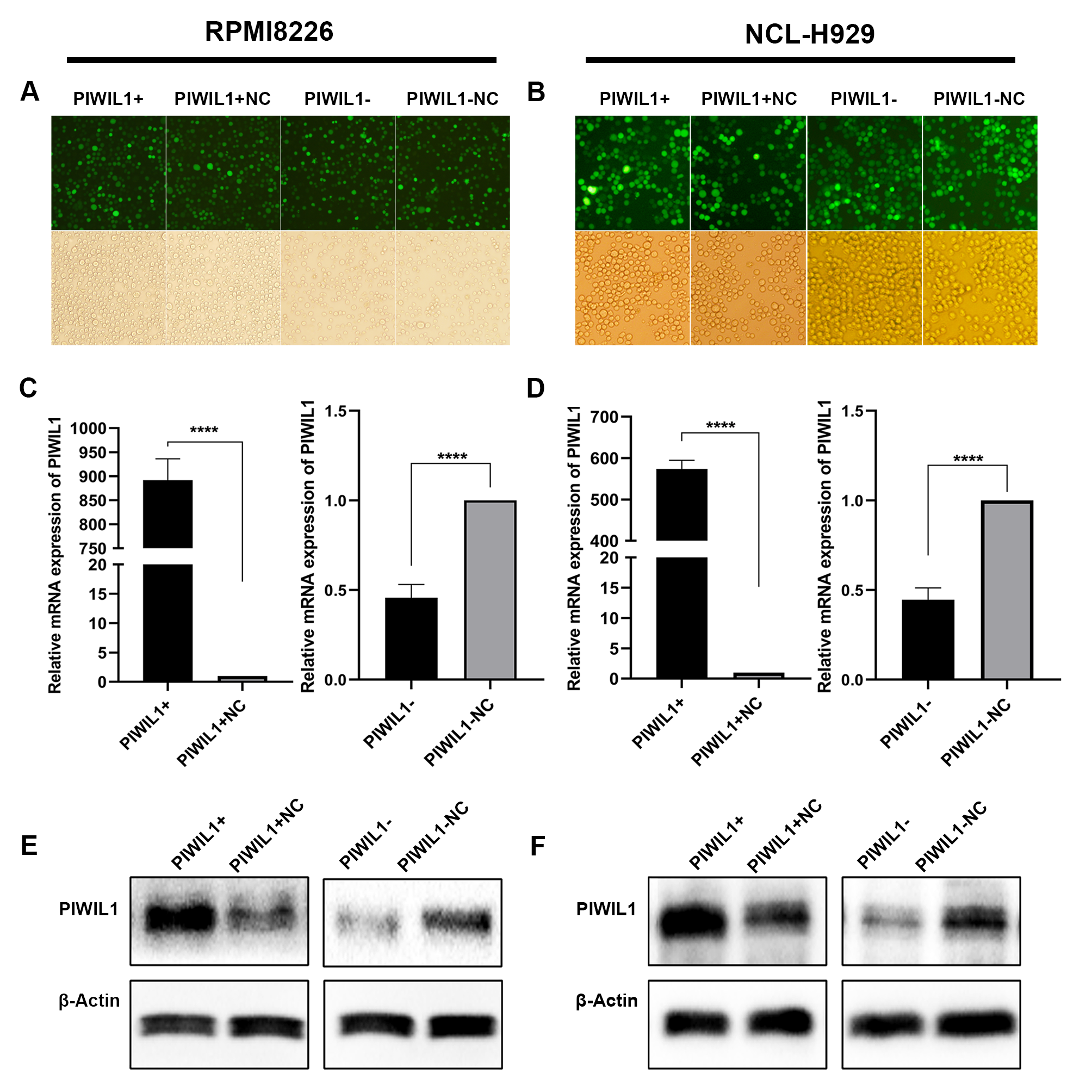

Supplement: Supplementary Figure 1 — Lentivirus transfection of RPMI-8226 and NCL-H929 cells. (A, B) MM cells (RPMI-8226 and NCL-H929) were transfected with a PIWIL1 or a shPIWIL1 lentivirus, and the transfection efficiency was assessed with a fluorescence microscope. (C, F) qRT-PCR (C, D) and western blot analysis (E, F) were used to verify the upregulation or downregulation of PIWIL1 in the lentivirus-transfected cells (n = 3). Data are from representative images or expressed as the means ± SEM of each group from three separate experiments (**** P < 0.0001). [file Image_1.tif]

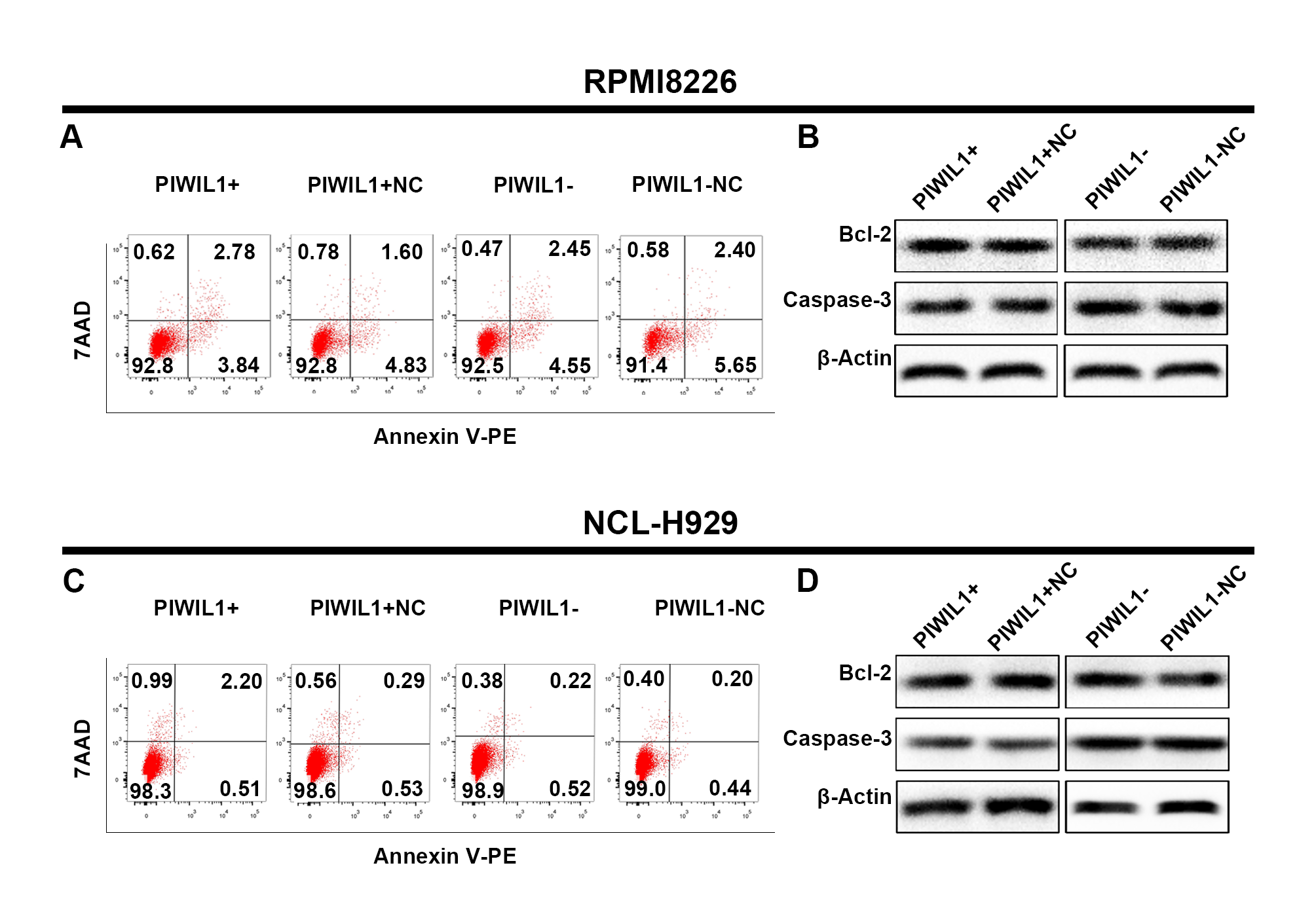

Supplement: Supplementary Figure 2 — PIWIL1 does not regulate apoptosis in MM cells. (A, C) Representative flow cytometry results showing apoptosis of MM cells (RPMI-8226 and NCL-H929) after PIWIL1 upregulation or downregulation. (B, D) Representative western blot images showing the expression of c-caspase-3 and BCL-2 after PIWIL1 upregulation or downregulation. Data are from representative images of each group from three separate experiments. [file Image_2.tif]

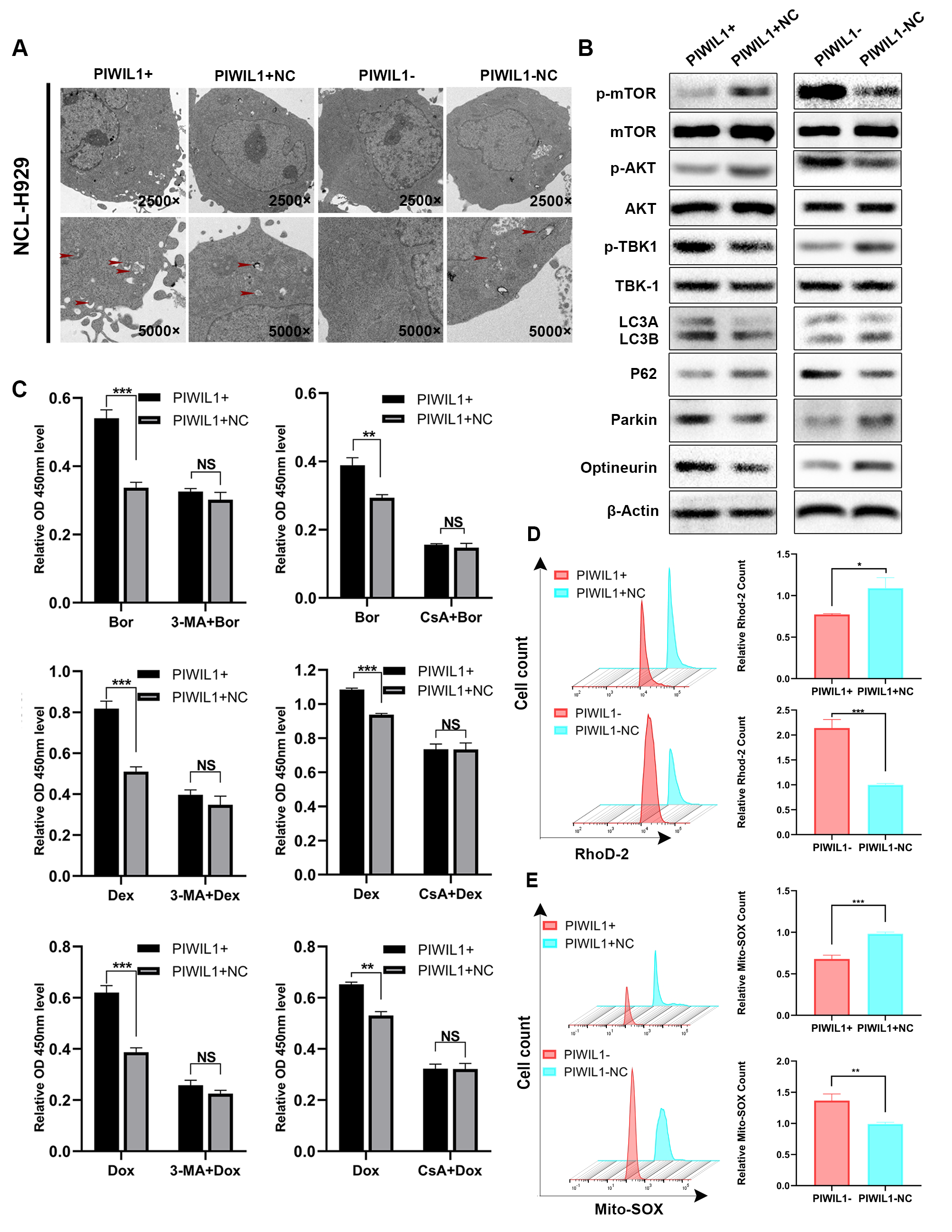

Supplement: Supplementary Figure 3 — PIWIL1 fosters drug resistance through autophagy and mitophagy in NCL-H929 cells. (A) Transmission electron microscopy of the morphological characteristics of NCL-H929 MM cells after transfection. Arrows indicate autophagosomes or autolysosomes. The panel shows the double-layered membrane engulfing a mitochondrion (early stage of mitophagy) and the double-layered membrane containing a degraded mitochondrion (late stage of mitophagy). (B) A representative result of the autophagy- and mitophagy-related protein levels detected by Western blot. (C) Different groups of transfected NCL-H929 MM cells were pretreated with the same dose of solvent, autophagy inhibitor 3-MA (5 mM) for 12 h or mitophagy inhibitor CsA (5 μM) for 24 h, followed by dexamethasone (100 μM), bortezomib (10 nM), and doxorubicin (25 nM) for 48 h. Cell viability of the different groups of transfected MM cells was assessed using the CCK-8 assay. (D) The left panel shows representative images of mitochondrial calcium changes in different groups. The right panel shows the mitochondrial calcium level presented as the relative MFI of Rhod-2 staining in NCL-H929 cells. (E) The left panel shows representative images of mitochondrial ROS level changes in different groups. The right panel shows mitochondrial ROS levels presented as the relative MFI of Mito-SOX staining in NCL-H929 cells. Data are from representative images or are expressed as the means ± SEM of each group from three separate experiments (**P < 0.01, NS, p > 0.05). [file Image_3.tif]

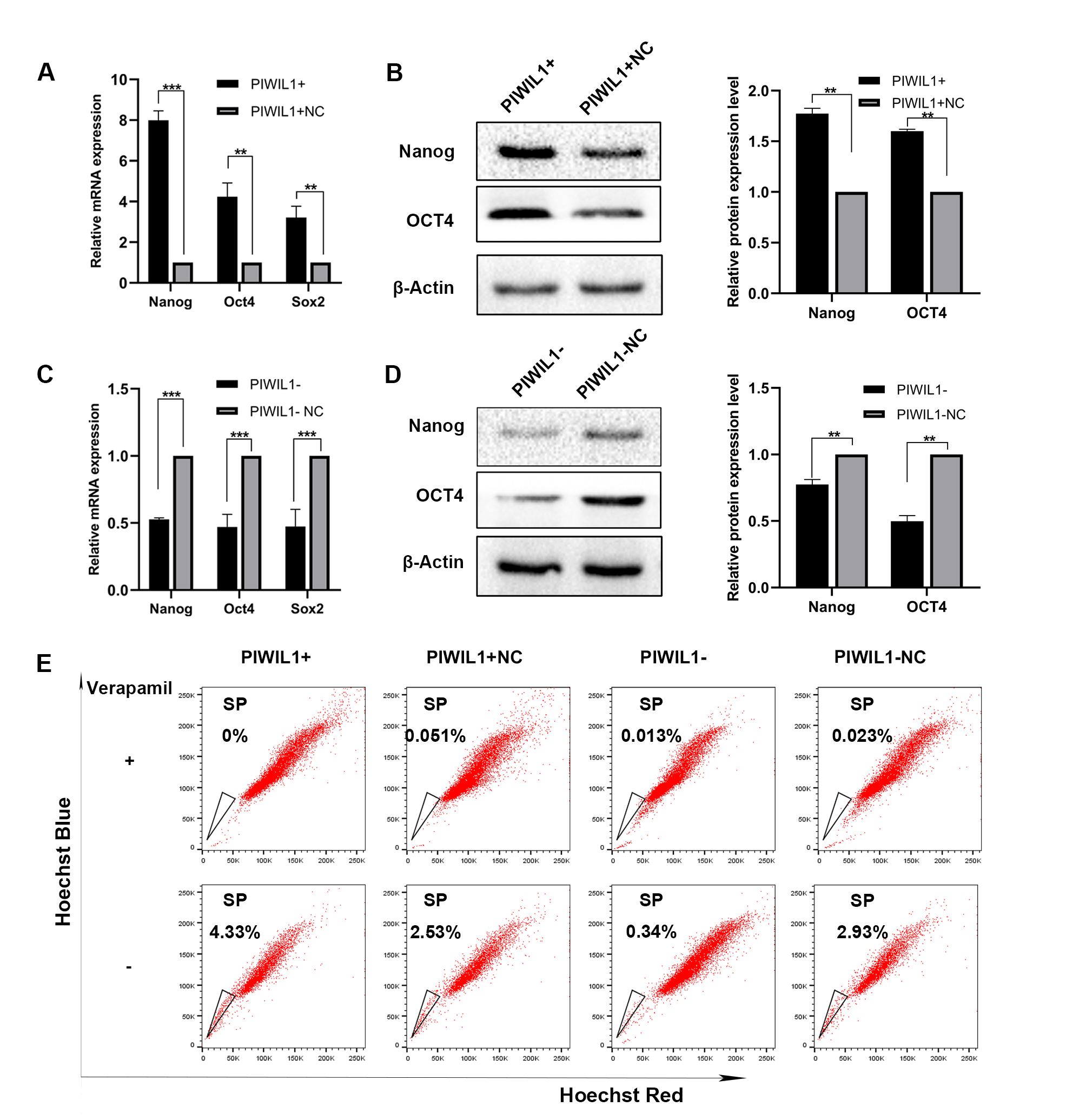

Supplement: Supplementary Figure 4 — PIWIL1 can regulate the stemness of NCL-H929 MM cells. (A) qRT-PCR results of the expression of stemness-related genes such as Nanog, SOX2, and OCT4 in different groups of transfected NCL-H929 MM cells. (B, D) Left panel: Western blot results of the expression of stemness-related proteins such as Nanog and OCT4 in different groups of transfected NCL-H929 MM cells; right panel: Histograms show the quantification of immunoblot images of Nanog and OCT4 proteins in MM cell as measured by ImageJ software. € Representative flow cytometric analysis of Hoechst-33342-based SP staining in transfected NCL-H929 cells. Data are from representative images or are expressed as the mean ± SEM of each group from three separate experiments (**P < 0.01, ***P < 0.001). [file Image_4.tif]

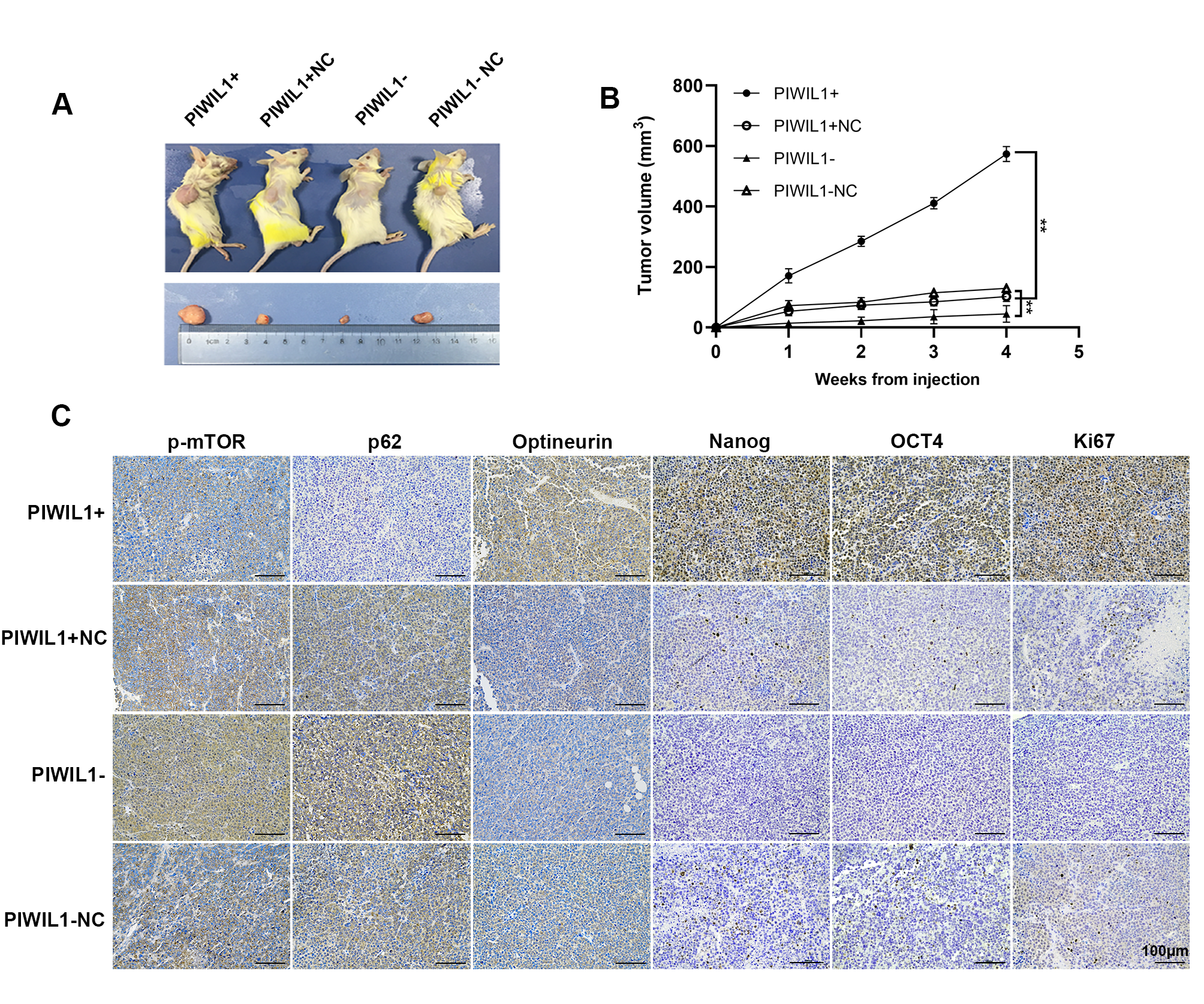

Supplement: Supplementary Figure 5 — PIWIL1 promotes the growth of xenograft tumors in vivo. (A) Tumor volume of NOD/SCID mice xenografted with NCL-H929 cells. (B) Tumor growth curves of NOD/SCID mice after injection with transfected NCL-H929 cells. (C) Representative immunohistochemical staining of p-mTOR, P62, optineurin, Nanog, OCT4, and Ki67 in tumor xenografts. Data are from representative images of three separate experiments (*P < 0.05, **P < 0.01). [file Image_5.tif]

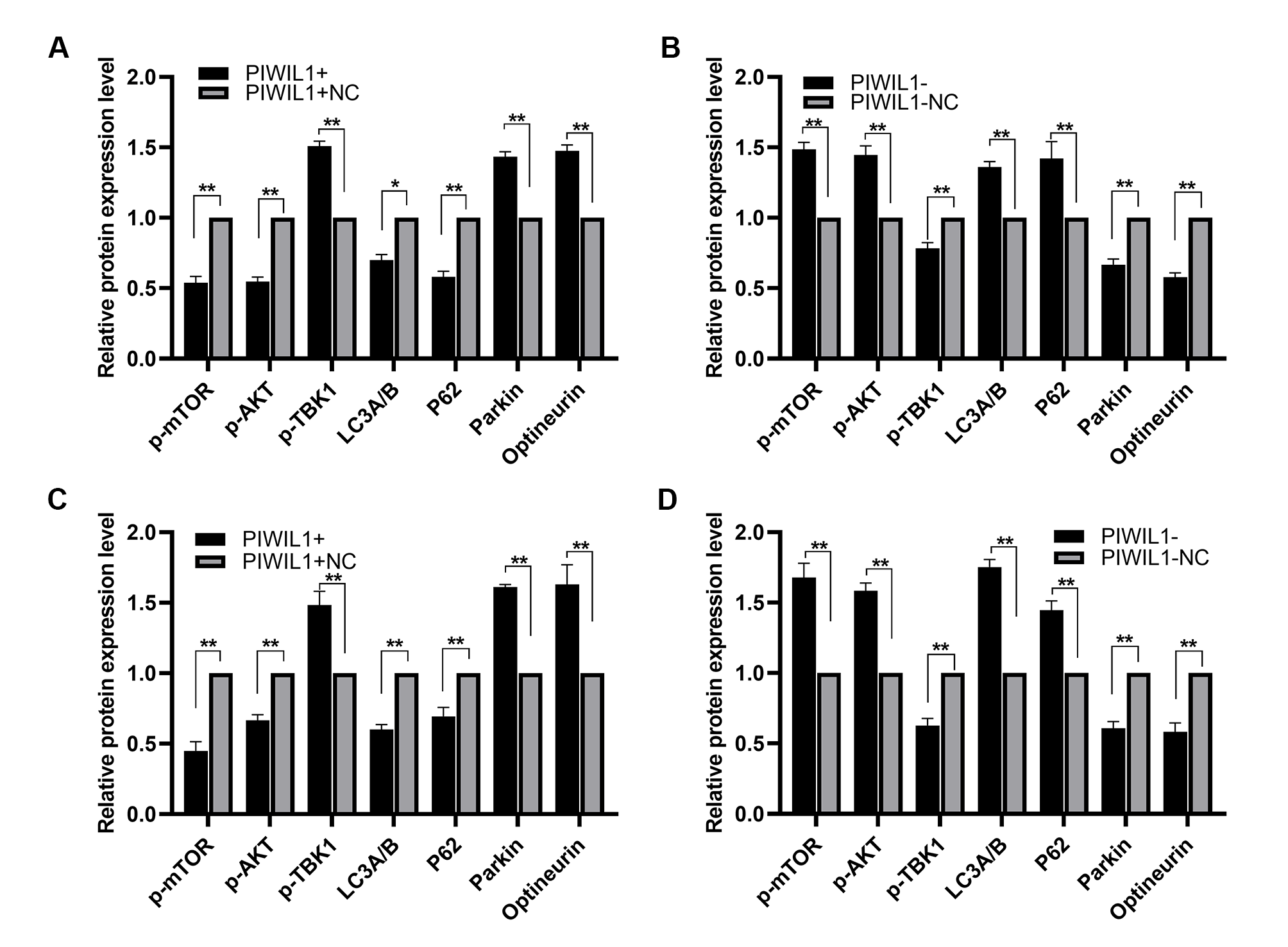

Supplement: Supplementary Figure 6 — Relative expression levels of autophagy-related proteins. (A, B) Histograms show the quantification of immunoblot images of autophagy-related proteins in transfected RPMI-8226 cells. (C, D) Histograms show the quantification of immunoblot images of autophagy-related proteins in transfected NCL-H929 cells. Data are from representative images or are expressed as the means ± SEM of each group from three separate experiments (*P < 0.05, **P < 0.01). [file Image_6.tif]
